# High-Fat Diet Induced Alteration of Mice Microbiota and the Functional Ability to Utilize Fructooligosaccharide for Ethanol Production

**DOI:** 10.3389/fcimb.2020.00376

**Published:** 2020-08-07

**Authors:** Rajnish Prakash Singh, Diana Abu Halaka, Zvi Hayouka, Oren Tirosh

**Affiliations:** Institute of Biochemistry, Food Science and Nutrition, Robert H. Smith Faculty of Agriculture, Food and Environment, The Hebrew University of Jerusalem, Jerusalem, Israel

**Keywords:** microbiota, fructooligosaccharide, fermentation, dietary fiber, ethanol

## Abstract

High-fat diet (HFD) leads to enhancement in various parameters of mice like weight, fasting glucose levels, adipose tissue, and also the liver weight in male C57 BL/6 J mice. Additionally, high-fat diet causes severe liver damage with significant increase in the level of aspartate amino transferase (AST) and alanine transaminase (ALT). The variations in microbiota induced by different diet were analyzed by Illumina MiSeq platform with sequencing of 16S ribosomal RNA (rRNA) gene, and QIIME pipeline was used. The population of Proteobacteria was found to be higher in HFD cecum sample as compared to other treatments. Microbiota analysis suggests that phylum Proteobacteria and Firmicutes were found to be higher in high-fat diet groups as compared to mice fed with normal diet (ND). At the genus level, *Bacteroides* showed higher population in HFD diet. Bacterial strains belonging to *Enterobacteriaceae* like *Escherichia, Klebsiella*, and *Shigella* were also dominant in HFD treatments. Furthermore, we explored the effects of ethanol production *in vitro* with supplementation of dietary fibers following inoculation of ND and HFD microbiotas. HFD microbiota of cecum and feces showed high level (*P* < 0.05) of ethanol production with 2% fructooligosaccharide (FOS) as compared to 2% galactomannan. Microbial fermentation also generated short-chain fatty acids (SCFAs), such as acetate, propionate, and butyrate. High levels (*P* < 0.05) of propionate were found after fermentation of FOS with HFD cecum and feces microbiota. The present study highlights the HFD-induced population of phylum Proteobacteria and genus *Bacteroides* for ethanol production using FOS as a dietary supplement, and these findings may imply on the harmful effect of HFD even at the microbiota level.

## Introduction

The gut microbiota comprises living microorganisms or microbiotas residing in the gastrointestinal (GI) tract of the host. Human health is widely affected by the composition and metabolic effect of these microbiota. The colonized microbiota population in the GI tract varies, with the large intestine having more density of microbes as well as the most metabolically active organ (Turnbaugh et al., 2006). The gut microbiota of humans and animals consists of 10^10^-10^14^ microbial cells that play a key role in the metabolic, immunological, and protective functions, which undoubtedly suggest the functionality of microbiota in health and disease (Goldsmith and Sartor, 2014). The recent developments in next-generation sequencing (NGS) technologies have allowed the detailed analysis of diverse gut microbiota and is presently being used as a tool to fill the gap on the microbiota studies using culture based methods. A human microbiome project lead by the National Institute of Health (NIH, USA) showed the presence of more than 70 bacterial phyla, with the majority belonging to Actinobacteria, Bacteroidetes, Firmicutes, and Proteobacteria. Similarly, another study of the gastrointestinal tract of rat microbiota revealed the presence of same predominant phyla (Actinobacteria, Bacteroidetes, Firmicutes, and Proteobacteria) in both cecum and fecal contents (Brooks et al., 2003). Therefore, using the NGS system for providing the number of studies on the gut microbiota leading to deep and accurate understanding is on the rise. These analyses showed that the gut microbiotas are closely linked with the stimulation of immune system and maintenance of the GI health (Kim et al., 2017). Among tested several factors, diet significantly influences the gut microbiota composition. Gut bacterial communities vary as per different components of the diet in a time-dependent manner. Therefore, recent studies have given priorities on the relationship between the diet and gut microbiota. Daniel et al. (2014) suggested that high-fat feeding leads to exceeding changes in the gut microbial community in mice, and these changes could be induced by increased microbial protein of amino acids in response to HFD. A recent *in vitro* study compared the microbiota in fecal samples after fermentation of different dietary fibers and estimated specific changes related to each individual fibers (Yang et al., 2013). Dietary fibers are complex polymers that are difficult to digest, which are being subjected to bacterial fermentation in the gastrointestinal tract, including the production of fermentative end products. Additionally, dietary fibers are heterogeneous in terms of degree of polymerization, origin, chemical composition, and physicochemical properties. Prebiotics are undigestible substances that confer beneficial effect on the host by stimulating the activity of indigenous bacteria (Slavin, 2013). Several prebiotics such as inulin, oligofructose, and fructooligosaccharide (FOS) showed the increase in fecal *Bifidobacteria* even at low consumption (5–8 g/day) (Slavin, 2013).

The intestinal microbiotas are highly metabolically active and are able to produce the ethanol. However, the increase in ethanol level play a role in the genesis of obesity-related fatty liver disease (Nosova et al., 1996). A small increase in the intestinal ethanol level leads to increased portal blood ethanol levels that induce the hepatic steatosis. Additionally, the increased ethanol level is responsible for redox changes that enhance the triglyceride accumulation in hepatocytes (Bujanda, 2000). The enhancement in ethanol level is also linked with enhanced risk of cirrhosis and liver disease. The other symptoms associated with ethanol could be the development of tumors, alterations in hormonal balance, and depletion of vitamin deposits (Bujanda, 2000). In the current study, we have monitored the ethanol levels after fermentation of FOS using the microbiota of ND and HFD. Microorganism within the Firmicutes phylum and LAB members comprising the genera like *Aerococcus, Enterococcus, Lactobacillus, Leuconostoc, Pediococcus, Streptococcus*, etc. are well-known for fermenting the sugars for ethanol production. Therefore, understanding the changes in the microbiome and subsequent changes to organic acid production in response to microbiome is of great interest.

The end products of fermentation, short-chain fatty acids (SCFAs), are produced through fermentation by intestinal microbiota using dietary fibers. Dietary fibers act as valuable energy sources for both cecum and colon-inhabiting bacteria of various groups. These microbiotas activate their metabolic pathways and machinery to metabolize the various chains of carbohydrates, which resulted in the production of metabolites like SCFAs. The major SCFAs produced are acetate, propionate, and butyrate (Pylkas et al., 2005); however, the level of produced SCFAs changes with the use of varying prebiotic sources. The produced SCFAs confer several cumulative effects on human health primarily acting as energy sources and also by minimizing the proliferation of deleterious bacteria of various groups (Pylkas et al., 2005). Ingestion of dietary fibers as prebiotic source in humans helps in the maintenance of metabolic profile, and the produced SCFA can reduce weight gain under high-fat feeding rodents. Among the SCFAs, acetate is the most abundant found in circulation and has been shown to cross the blood–brain barrier (Perry et al., 2016). Butyrate is primarily used as an energy source for colonocytes and enterocytes. Propionate is utilized locally through conversion into glucose by gluconeogenesis processes in the intestine. Propionate may also enter in the circulation to be utilized for hepatic gluconeogenesis (De Vadder et al., 2014). Propionate commonly acts as a neoglucogenic substrate for the liver and has been well-known for increasing the adipogenesis as well as inhibiting the lipolysis in the adipose tissue of mice. Being an important molecule for counteracting the cholesterol synthesis, the ratio of acetate/propionate plays a key role in regulating the cholesterol and lipid metabolism (Wong et al., 2006).

In the current study, we aimed to investigate the impact of normal and high-fat diet on the gut microbiota populations. High-throughput sequencing technology was used to provide the changes in gut microbiota in response to a HFD and ND. To gain insights into ethanol production, the control and high-fat diet microbiota of fecal and cecum were tested with different dietary fibers. Taken together these observations and emphasizing the important role of SCFAs in immune regulation, the influence of microbiota of the cecum and feces on the SCFA production was determined using the selected dietary fiber FOS. The current research provides microbial mechanistic insights of ethanol production using FOS as dietary fibers.

## Materials and Methods

### Ethics and Permission

Animal experiments were done in accordance with relevant guidelines and regulation and approved by the Institutional Animal Care Ethics Committee, Hebrew University of Jerusalem.

### Animal and Diets

C57BL/6J is a well-known commercially available mice strain that is an acceptable model for diet-induced obesity and liver damage (Harlan Laboratories, Jerusalem, Israel). Seven- to eight-week-old male mice (*N* = 5) were randomly assigned to two groups, one fed with a normal diet (ND) and other with high-fat diet (HFD). Mice were housed in sterile cages (three to four mice/cage) in a pathogen-free animal house with a temperature of 22 ± 2°C and relative humidity of 50 ± 10%, under 12-h light/dark cycle. The low- and high-fat composition has been summarized in Supplementary Table S1.

After 8 weeks on the diets, the mice were fasted for 12 h and sacrificed in random order by isoflurane overdose. Before the sacrifice, body weights of each mice in tested groups were recorded. The epididymal adipose tissue was stored at −75°C. A small portion of the liver tissue was placed in formaldehyde solution of 4% concentration, and the remaining liver tissue was stored at −80°C for further tests. Similarly, intestinal tissues were collected and frozen for further experimental purposes. The cecum was separated from the large intestines, and its contents were immediate processed for microbiota analysis. Similarly, feces were collected from each group (ND and HFD) and further processed for microbiota analysis. Additionally, live damage markers aspartate aminotransferase (AST) and alanine aminotransferase (ALT) were also tested as per standard protocol.

### Fecal and Cecal Bacterial DNA Extraction

Fecal and cecal samples were collected from individual mice in autoclaved tubes and stored at −80°C. Before storage, they were frozen in dry ice. Epicenter DNA isolation kit was used for isolating the DNA (five replicates in each dietary group) following the protocol's instruction. DNA concentration and purity were then determined with a Nano-Drop 2000 UV–Vis spectrophotometer (Thermo Fisher Scientific, USA). All metagenomic DNA samples were stored at 4°C for microbial community analysis.

### Analysis of Microbial Community

Genomic DNA was extracted, and PCR was performed with primers CS1_515F and CS2_806R (Parada et al., 2016; Walters et al., 2016) targeting the V4 regions of microbial ribosomal RNA genes (small subunit) following the standard protocol of targeted amplicon sequencing (Bybee et al., 2011; Naqib et al., 2018). PCR amplifications were performed in a final volume of 10 μl reactions using the MyTaq HS 2 × Master Mix with conditions at 95°C for 5 min, followed by 28 cycles of 95°C for 30 s, 55°C for 45 s, and 72°C for 30 s.

Afterwards, a second PCR amplification was performed with a separate primer pair with a unique 10-base barcode in 96-well plates. Each well-received were obtained from the Access Array Barcode Library for Illumina (Fluidigm, South San Francisco, CA). Library preparation, pooling, and miniSeq sequencing were performed at the University of Illinois at Chicago Sequencing Core (UICSQC). The reads of both forward and reverse ends were combined using PEAR (Zhang et al., 2014). After merging, reads were trimmed to remove the sequences <225 bp. USEARCH algorithm was used to remove the chimeric sequences. Furthermore, taxonomic summaries were generated with the standard QIIME pipeline (Caporaso et al., 2010; Tikhonov et al., 2015). USEARCH and Silva v132 reference were used for taxonomic annotations for seed and unmatched non-seed sequence to obtain a minimum threshold of 90% (Edgar, 2010; Glöckner et al., 2017).

### Microbial Culturing and Ethanol Production

The fecal and cecal samples of ND and HFD were processed into the nutrient broth medium for enrichment of culturable bacteria. To screen the optimum ethanol production, basal minimal medium (MM) containing 64 g/L Na_2_HPO_4_.7H_2_O, 15 g/L KH_2_PO_4_, 5 g/L NH_4_Cl, and 2.5 g/L NaCl with 2 mM MgSO_4_ and 0.1 mM CaCl_2_ was autoclaved (121°C, 15 min) and allowed to cool down at room temperature and was further supplemented with dietary fibers (2% FOS and 2% galactomannan). Bacterial cultures of fecal and cecum samples of both normal and high-fat diet were diluted to get 1 ×10^7^ cells and mixed with minimal medium supplemented with 2% of dietary fibers. The culture was incubated for 24 h at 37°C temperature with shaking at 200 rpm. After the incubation period, the medium in each treatment was filtered with 0.22-μm Millipore filters and run on gas chromatography (GC) to measure the ethanol production. The column used was HP-FFAP (Agilent Technologies, USA) equipped with a capillary column (30 m ×0.53 mm ×1.0 μm, Agilent Technologies, USA) and a flame ionization detector (FID). The injector and detector temperature were set at 200 and 240°C, respectively. The initial GC oven temperature of 120°C was held for 1 min and then ramped at 10°C/min to a final temperature of 240°C. The total run time for GC was 13 min/sample. The peaks were identified by comparing the retention time with the standard area. Calibration curves were plotted, and the standard showing *R*^2^ value close to 0.999 was accepted. The standard curve of ethanol was generated with 0.1–0.5% of 95% ethanol.

### Short Chain Fatty Acids (SCFAs) Analysis

The acetic, propionic, and butyric acid production in the fermentations was determined by GC (Merck, USA) equipped with RI detection. One-milliliter aliquots from each treatment in the above experiment were centrifuged at 10,000 g for 10 min to remove the particulate matter. The obtained supernatants were filtered with 0.22-μm polyvinylidene fluoride (PVDF) membranes (Millipore, USA). Following filtrations, 1 μl of each sample was injected for the 13-min run. Integration of peaks was performed with Atlas Lab Managing Software (Thermo Labs, Germany). The tested samples were quantified through calibration curves of acetic, propionic, and butyric acids in concentration ranging between 0.1 and 0.5%.

### Enumeration of Microorganisms After Fermentation

During the time course of fermentation, 1 ml of each sample was serial diluted (1:10) into phosphate-buffered saline solution (KCl, 2.7 mM; KH_2_PO_4_, 1.8 mM; Na_2_HPO_4_, 8.1 mM; NaCl, 0.14 M; pH 7.4). Finally, 100 μl of appropriate dilutions was plated on the Luria–Bertani (LB) agar media, and plates were incubated at 37°C for 24–48 h. The colonies observed on the plate were counted and reported as CFU/ml.

### Statistical Analysis

Ethanol production and SCFAs were statistically analyzed, and values are presented as means ± SEM. Means were compared with analysis of variance (one-way ANOVA) followed by the *t*-test using the significant level of *P* < 0.05 for all analyses. The analysis was performed with the JMP 7.0.2 and JMP Pro software suites (SAS Institute, USA). The correlation coefficient was used to analyze correlation between the abundance of phylum Proteobacteria and genus *Bacteroides* in ND and HFD and ethanol production.

## Results

### Comparing Physiological Parameters of ND and HFD Mice

**Figure 1:**
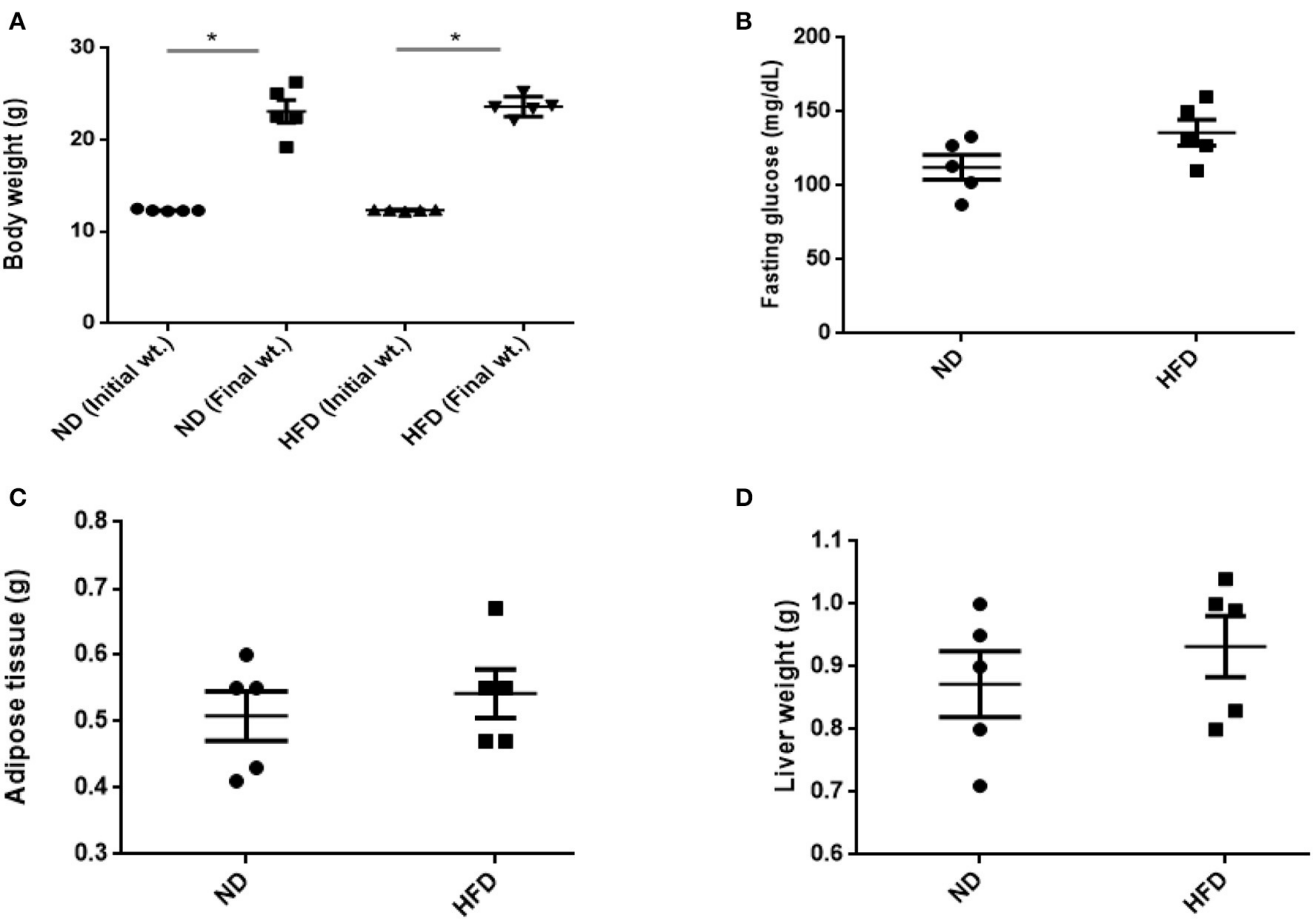
Effect of normal diet (ND) and high-fat diet (HFD) feeding for 8 weeks on various parameters: **(A)** body weight, **(B)** fasting glucose level, **(C)** weight of adipose, and **(D)** liver tissue. All values are mean ± SEM (*n* = 5). Columns marked with asterisk are significantly different (*P* < 0.05) to its counterpart, whereas symbol ns represents the no significant difference.

**Figure 2:**
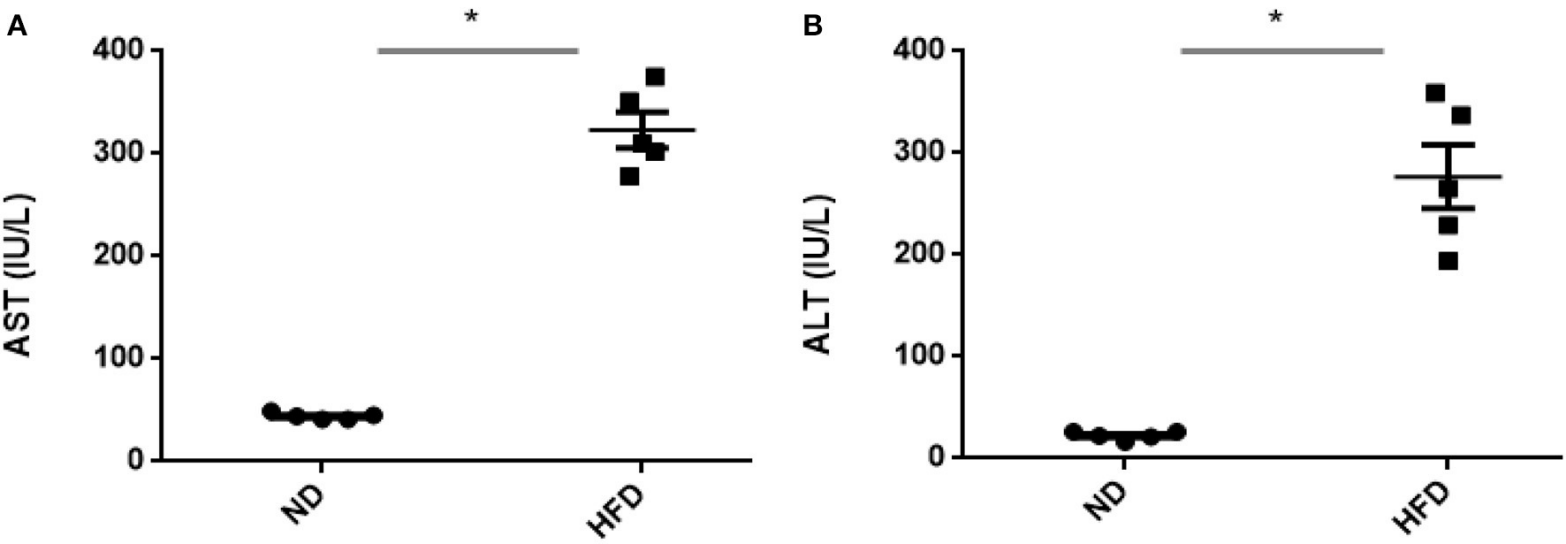
Assessment of liver damage after high-fat diet (HFD) feeding for 8 weeks by measuring aspartate **(A)** transaminase and **(B)** alanine transaminase. All values are mean ± SEM (*n* = 5). Asterisk has been used for significant differences (*P* < 0.05).

To evaluate obesity traits responding to diet, male C57BL/6J mice were fed with ND and HFD for 8 weeks. Both groups gain weight during the experiment, but we could not observe significant weight gain (*P* > 0.05) when compared with ND-treated mice to HFD supplemented with cholic acid (Figure 1A). As compared to ND treatment, fasting glucose was higher (147.6 mg/dl) in the HFD-treated mice; however, in ND treatment, the level of fasting glucose was 112.4 mg/dl (*P* > 0.05; Figure 1B). On the day of sacrifice, epididymal adipose, and liver tissues were removed and weighed. As compared to ND treatment, the weights of the adipose tissue and liver were slightly higher by 9% (*P* > 0.3931) and 16.7% (*P* > 0.05) in HFD treatment (Figures 1C,D). Moreover, HFD mice showed significant increase in AST (*P* < 0.001) and ALT (*P* < 0.001) levels as compared to ND, illustrating the liver injury in HFD treatments (Figure 2).

### Microbial Diversity and Composition

**Figure 3:**
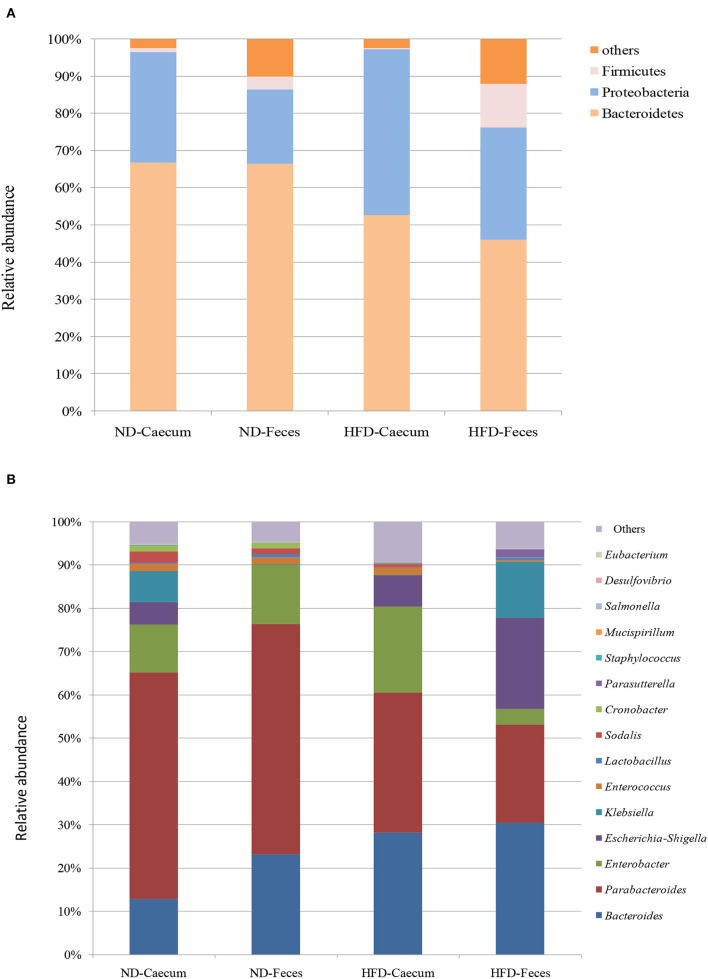
Effects of high-fat diet (HFD) enrichment on changing microbiota populations was evaluated in cecum and feces of normal diet (ND) and HFD-treated mice after 8 weeks. **(A)** Relative abundance (%) of most prevalent bacteria at phylum level. **(B)** Relative abundance (%) of most prevalent bacteria at genus level.

The microbiomes of HFD- and ND-treated mice (cecum and feces) were sequenced by high-throughput Illumina MiniSeq. Sequencing of 16S rRNA V4 gene generated 546,676 reads, with an average of 31,849 reads per sample. Furthermore, quality filtering resulted in removal of 1.2% of reads across all samples, tested by quality control procedures. The ultimate goal of the present study was to identify the differences in microbial composition between the HFD and ND groups. The most abundant bacteria belong to the phyla Bacteroidetes, Proteobacteria, and Firmicutes (Figure 3A). Bacterial taxa of each class (HFD and ND) were identified and characterized by genera *Parabacteroides, Bacteroides, Enterococcus, Lactobacillus, Cronobacter, Sodalis, Parasutterella, Escherichia, Shigella, Klebsiella*, and *Enterobacter* in addition to some undefined members of families *Tannerellaceae*. Most of these genera were found to be differentially distributed among HFD and ND groups (Figure 3B). By considering the most representative taxa, the overall microbiota consisted of 6 phyla (Actinobacteria, Bacteroidetes, Deferribacteres, Firmicutes, Proteobacteria, and Tenericutes), 11 classes (Actinobacteria, Coriobacteria, Bacteroidia, Deferribacteres, Bacilli, Clostridia, Erysipelotrichia, Alphaproteobacteria, Deltaproteobacteria, Gammaproteobacteria, and Mollicutes), 19 orders (Bifidobacteriales, Corynebacteriales, Micrococcales, Coriobacteriales, Bacteroidales, Chitinophagales, Cytophagales, Flavobacteriales, Deferribacterales, Lactobacillales, Clostridiales, Erysipelotrichales, Bdellovibrionales, Desulfobacterales, Betaproteobacteriales, Enterobacteriales, Anaeroplasmatales, Entomoplasmatales, and Mollicutes), 25 family (*Bifidobacteriaceae, Corynebacteriaceae, Eggerthellaceae, Bacteroidaceae, Prevotellaceae, Rikenellaceae, Tannerellaceae, Microscillaceae, Crocinitomicaceae, Deferribacteraceae, Bacillaceae, Staphylococcaceae, Enterococcaceae, Lactobacillaceae, Leuconostocaceae, Streptococcaceae, Clostridiaceae, Lachnospiraceae, Ruminococcaceae, Erysipelotrichaceae, Desulfobacteraceae, Burkholderiaceae, Enterobacteriaceae*, and *Anaeroplasmataceae*), and 27 genera (*Bifidobacterium, Corynebacterium, Enterorhabdus, Bacteroides, Bacteroidaceae, Odoribacter* (*Parabacteroides, Tannerellaceae, Enterococcus, Lactobacillus, Lactococcus, Streptococcus, Clostridium, Lachnospiraceae, Ruminiclostridium, Ruminococcaceae, Dubosiella, Faecalibaculum, Bilophila, Desulfovibrio, Parasutterella, Burkholderiaceae, Enterobacter, Escherichia–Shigella, Klebsiella*, and *Salmonella*). Microbiota associated to HFD cecum showed a higher relative abundance of Proteobacteria (44.5%), as compared to other treatments (25–30%).

Overall taxa analysis showed a significant (Kruskal–Wallis *P* = 0.039) increase in the ratios of *Bacteroides* among ND and HFD feces of mice. Among the gut-dominating bacterial phylum Firmicutes, its population was higher in HFD feces (12.8%) compared to that in ND feces (3.4%) and ND cecum (1%). Similarly, Proteobacteria were found to be higher in HFD cecum (44.5%), as compared to other treatments. Core bacterial taxa analysis suggests that *Bacteroides* (35.39%) were abundant in HFD feces of mice as compared to other treatments. *Parabacteroides* population were more abundant in ND cecum (52.32%) and ND feces (35.6%) followed by HFD cecum (33.17%) and HFD feces (21%) samples. Interestingly, bacterial strains belonging to *Enterobacteriaceae*, like *Klebsiella*, and *Enterobacter* population, were found to be differentially distributed. At genus level, HFD feed had higher abundance of *Enterobacter* (20.5%), *Escherichia–Shigella* (19.5%), *Klebsiella* (12.12%), and *Parasutterella* (1.90%).

It revealed that genus *Bacteroides* and *Enterobacteria* were clearly enriched in high-fat diet cecum and feces. Other minor groups were also significantly abundant in the high-fat diet treatments. Furthermore, we examined the correlation between relative abundance of Proteobacteria and *Bacteroides* in ND and HFD treatment and ethanol production. A positive correlation (*R*^2^ = 0.139, *P* = 0.0780) was found between the ethanol production and relative abundance of *Bacteroides* in the ND and HFD cecum (Supplementary Figure S1A). Similarly, the proportions of *Bacteroides* in ND and HFD feces showed a positive relationship (*R*^2^ = 0.167, *P* = 0.1049) with ethanol production (Supplementary Figure S1B). In addition, the relative proportion of phylum Proteobacteria of ND and HFD cecum showed positive relationship (*R*^2^ = 0.424, *P* = 0.0403) with ethanol production (Supplementary Figure S2A). In the phylum Proteobacteria, a positive correlation (*R*^2^ = 0.495, *P* = 0.022) was found between the ethanol production and relative abundance of Proteobacteria in ND and HFD feces (Supplementary Figure S2B).

### Ethanol Production

**Figure 4:**
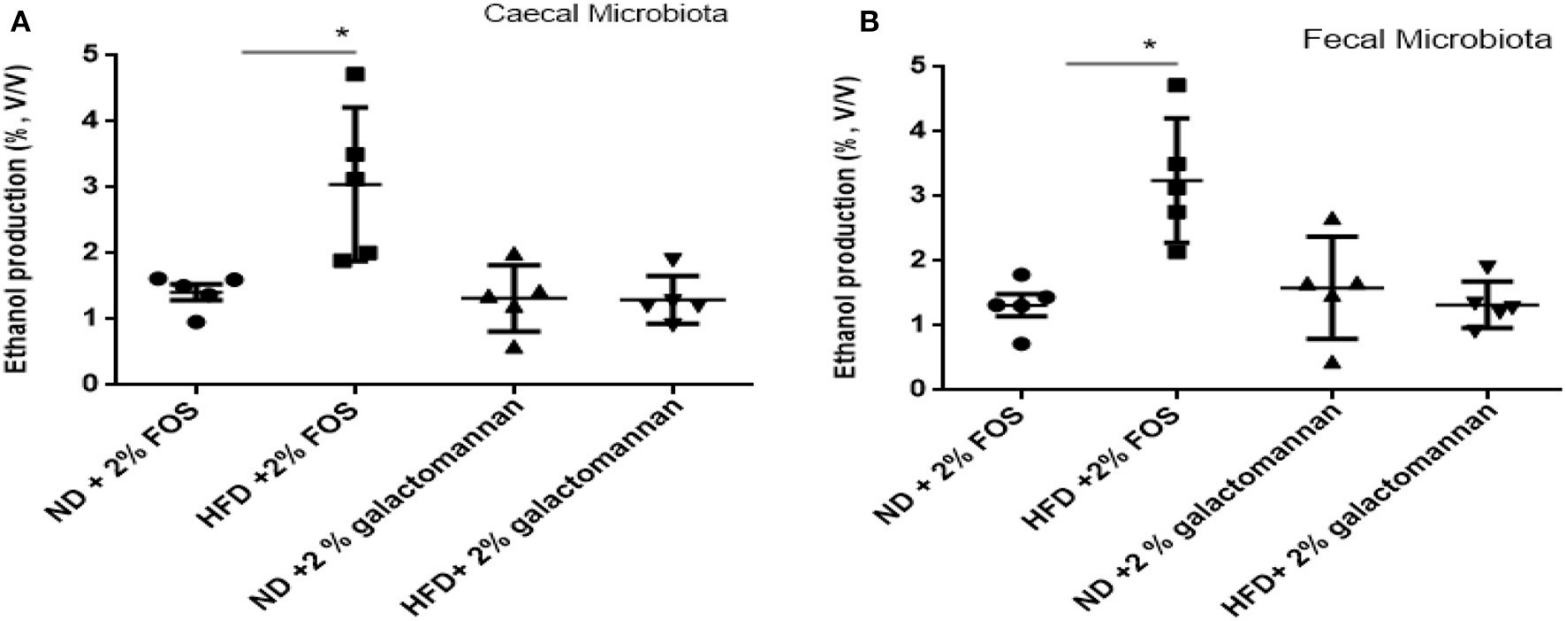
Effects of normal diet (ND) and high-fat diet (HFD) microbiota inoculation on ethanol production with fructooligosaccharide and galactomannan. **(A)** Cecum microbiotas of ND and HFD were tested for ethanol production. **(B)** Measurement with fecal microbiota of ND and HFD. All values are mean ± SEM (*n* = 5). Significant difference (*P* < 0.05) among treatments has been shown by asterisk.

The microbiomes of ND and HFD were tested for ethanol production ability when fermented with different dietary fibers. As shown in Figure 4A, higher ethanol production was observed when FOS was used as a substrate as compared to galactomannan in the same concentration. As compared to control (ND) microbiota, significant (*P* < 0.05) ethanol production (3%) was observed when FOS were utilized by the HFD cecum microbiota. Both control and HFD microbiota were able to produce 1.08–1.20% ethanol when galactomannan was used as a substrate. Similarly, significant enhanced level of ethanol (3.40%) was observed by HFD fecal microbiota (Figure 4B).

### SCFA Production

**Figure 5:**
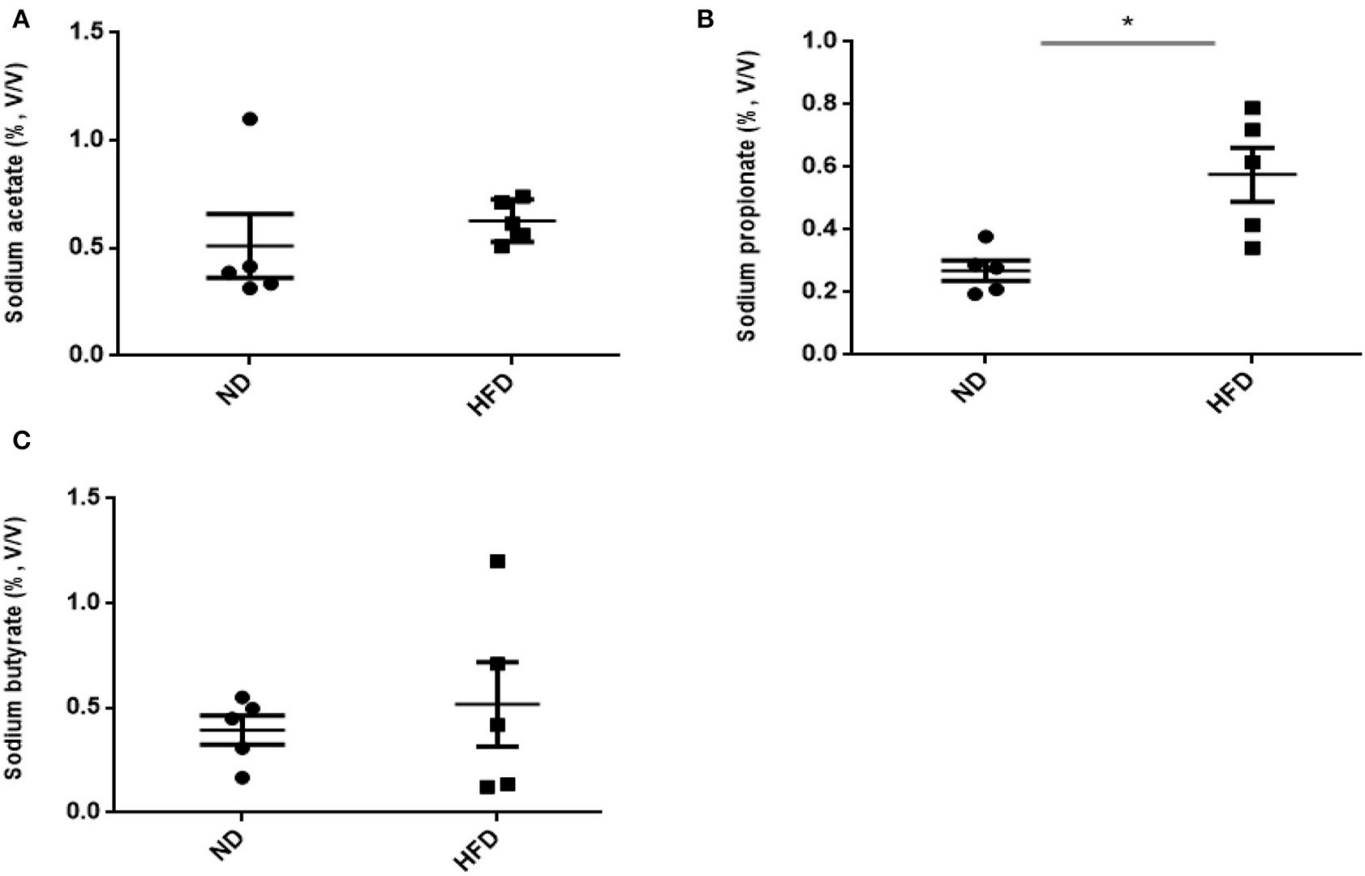
Measurement of short-chain fatty acid (SCFA) production after treatment of fructooligosaccharide with normal diet (ND) and high-fat diet (HFD) fecal microbiota. **(A)** Sodium acetate, **(B)** sodium propionate, and **(C)** sodium butyrate. Results are means ± SEM (*n* = 5). Asterisk represents significant difference (*P* < 0.05) among tested treatments.

**Figure 6:**
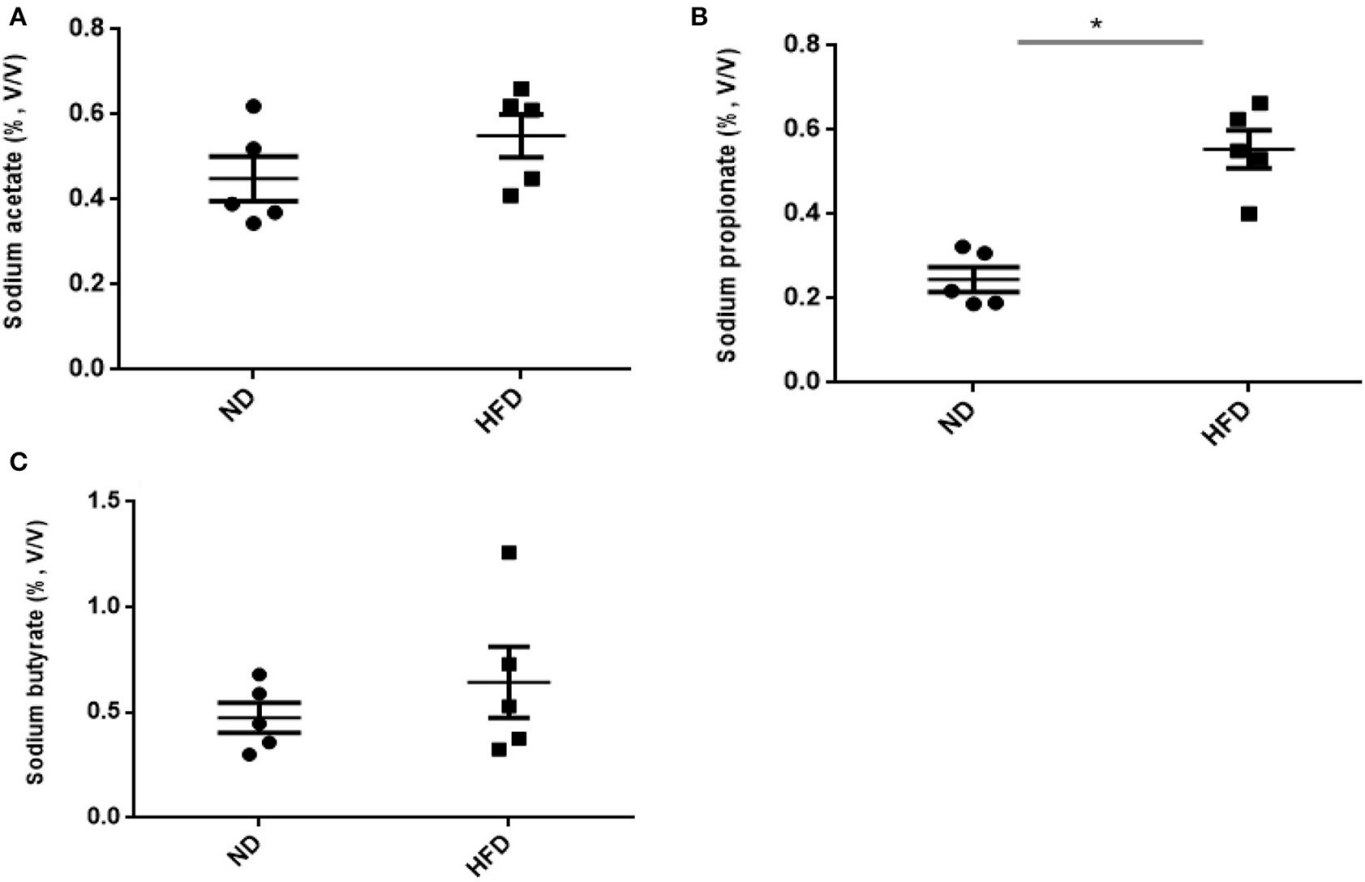
Measurement of SCFA production after treatment of fructooligosaccharide with normal diet (ND) and high-fat diet (HFD) cecum microbiota. **(A)** Sodium acetate, **(B)** sodium propionate, and **(C)** sodium butyrate. Results are means ± SEM (*n* = 5). Asterisk represents the significant difference (*P* < 0.05) among tested treatments.

In addition to ethanol production, we also quantified the SCFA production in the presence of FOS. The results of SCFA are in Figures 5, 6. The fermentation of FOS by ND and HFD microbiota illustrates the significant (*P* < 0.05) enhancement in the sodium propionate (51.6%) in response to HFD fecal microbiota. However, there was little change in sodium acetate (14%) and sodium butyrate (22%). Regarding the fecal microbiota, HFD cecum microbiota also showed significant (*P* < 0.05) increase in the sodium propionate (44%), whereas there was little change in sodium acetate (20%) and sodium butyrate (24%) (Figure 6).

### Bacterial Population Count

The change in bacterial number in terms of colony forming units was recorded at different times (6, 12, 18, and 24 h) during fermentation with FOS in both ND and HFD treatments. As shown in Supplementary Figure S3, the bacterial number increases with respect to time in both cecum (Supplementary Figure S3A) and fecal (Supplementary Figure S3B) tested microbiota. Initially, there were 1 ×10^7^ CFU/ml of bacteria in both ND and HFD; however, after 24 h of fermentation, the loading of HFD microbiota reached up to 1.40 ×10^12^ CFU/ml.

## Discussion

The present results showed that enrichment with HFD in mice leads to an increase in the level of liver damage markers AST and ALT, which illustrate severity of liver damages in response to high-fat diet. These findings were consistent with the previous results that showed severe liver damages while feeding mice with high-fat diet (Murphy et al., 2013; Boursier and Diehl, 2015). Enrichment with HFD led to increase in body weight and glucose level; however, the levels did not significantly differ as compared to ND. Similarly, the adipose tissue and liver weights were slightly increased in HFD mice.

In the present study, C57BL/6J mice were chosen to compare the microbiota of ND and HFD of cecum and feces. There are few studies that showed the manipulation of gut microbiota under HFD; however, in our study, we also investigated the effects of microbiome on the fermentation of different dietary fibers and SCFA production. Enriching the diet with 60% fat was found to modulate gut microbiota and enhance the diversity of the gut microbiota. We sought to explore the microbiome of HFD-treated mice in comparison with healthy control diet treatment. Hence, we sequenced and analyzed the microbial community structure in 10 HFD mice and 10 healthy controls and observed few differences. At the phylum level, HFD enhanced the abundance of species belonging to phylum Firmicutes and decrease in Bacteroidetes, which is in congruence with other studies (Turnbaugh et al., 2006). A previous study (Huang et al., 2013) suggested that ingestion of unsaturated fat (74%) elevated the level of microbiota of various groups like Firmicutes, *Proteobacteria*, etc. as compared to low-fat diet. Genera *Bacteroides, Escherichia, Klebsiella*, and *Enterobacter* were more enriched in HFD-treated mice, and *Parabacteroides* were more abundant in normal diet mice. In congruence to other microbiota studies, a higher microbial diversity was observed in HFD-treated samples as compared to controls. Interestingly, HFD, which is rich in oleic acid, in the present study also induced the population of *Proteobacteria* mostly resulting from an increase in the order Desulfovibrionales.

Bacterial family like *Bacteroidaceae* and *Enterobacteriaceae* were the most common associated with the HFD. Bacterial taxa belonging to these families are commonly associated with the gut health maintenance and also act as active degraders of plant-derived material in the gut (Biddle et al., 2013). These observed results are consistent with other findings where microbiotas belonging to the Bacteroidetes are responsible for multiple important roles including metabolites generation, maintenance of immune system, angiogenesis, etc. (Semova et al., 2012). The relative abundance of Bacteroidetes and Firmicutes phyla varies depending on food source, with a higher relative proportion of Firmicutes being associated with HFD. Koliada et al. (2017) has shown that HFD-treated human and animal organisms have enhanced ratio of Firmicutes/Bacteroidetes as compared to normal diet. However, a recent study by Dalby et al. (2017) has demonstrated that changed ratio of Firmicutes/Bacteroidetes in mice has no relation with high-fat presence, and it is solely related to presence of dietary fiber contents. Similarly, previous studies showed the impacts of dietary behavior on gut microbiota modulation and suggested that HFD feeding leads to increase in the Bacteroidetes (Wu et al., 2011; Clarke et al., 2012). Few studies in humans and rats demonstrated an increase in the population of Bacteroidetes in response to an HF diet (Angelakis et al., 2012). Increased level of Proteobacteria population serves as a potential diagnostic marker for dysbiosis and other metabolic disease (Shin et al., 2015). In the current study, the higher level of Proteobacteria was found in the HFD treatments.

In relation to functional activity of the microbiome, it has been reported that the HFD-associated gut microbiota has higher efficiency of metabolic processes through colonic fermentation and metabolites like SCFA production (Turnbaugh et al., 2006). The assimilation of produced SCFAs into alternative energy sources might enhance the level of cholesterol and also boost the gluconeogenesis processes. Mattison et al. (2014) showed that HFD treatment leads to changes in the gut-associated microbiota population. In congruence to a previous study, we found that diet enriched with high-fat content leads to increased Proteobacteria and Firmicutes and decreased Bacteroidetes. Bacteroidetes and Parabacteroides have been reported to produce SCFAs by the fatty acid biosynthesis pathway (Zeng et al., 2017).

In the present study, we observed elevated acetate level via microbiome of HDF-treated mice, which was consistent with previous reports. A previous study by Koh et al. (2016) has reported that SCFAs are generally produced from the microbial fermentation of dietary fibers, and its level can be enhanced by the intake of HFD (Perry et al., 2016). Therefore, at present, it is estimated that, under HFD treatment, SCFA levels are affected only by fiber fermentation. Similarly, our study showed that there was also enhanced level of sodium propionate and sodium butyrate in HFD treatment. Among SCFAs, the higher level of produced propionate acts as neurotoxic and metabolic toxin in liver cells by accumulating in mitochondria as propionyl-CoA. Aside from this, it also affects various metabolic processes such as gluconeogenesis, ureogenesis, or ketogenesis (Remsy et al., 1992). SCFA analysis revealed slight enhancement in acetate and butyrate production in response to aHFD. The elevated SCFA level in HFD-treated mice can affect colonic pH and also alter the microbial community (den Besten et al., 2013). Additionally, SCFAs also influence the motility and intestinal immunity, regulate G-protein-coupled receptors, and provide fuel for colonocytes (Donohoe et al., 2011). Therefore, exploring the changes in the microbiome and subsequent changes to organic acid production under HFD treatment is of great interest.

## Conclusion

The present study revealed that diet with different fat compositions and bile acids showed severe liver damages, with certain changes in physical physiological parameters in mice. Gut microbiota population was changed under different diet feeding, and bacterial richness and diversity in HFD were more as compare to ND even without weight gain. The relative abundance of bacteria related to Proteobacteria and Firmicutes was higher in HFD treatments. The higher production of ethanol by HFD feces/HFD cecum microbiota with fructooligosaccharide underlies the role of Proteobacteria and Firmicutes in fermentation. The higher abundance of *Bacteroides* in HFD cecum/feces emphasizes its role in ethanol production. Similarly, the higher production of deleterious SCFA by HFD microbiota emphasizes the most appropriate parameter to identify the microbiota-regulating effect of diet and also the importance to understand the responses to a prebiotic treatment for mechanistic understanding and human application. However, *in vitro* culturing being a timely and cost-efficient way to discover microbiota responses to different dietary fibers, it may not maintain the functional and compositional profiles of gut microbiome. Therefore, future studies should focus on *in vivo* microbial ecosystem as shifts in microbiome composition can alter their functional properties.

## Data Availability Statement

The datasets presented in this study can be found in online repositories. The names of the repository/repositories and accession number(s) can be found in the article/Supplementary Material.

## Ethics Statement

This animal study was reviewed and approved by Institutional Animal Care Ethics Committee, Hebrew University of Jerusalem.

## Author Contributions

RS: conceptualization, methodology, analysis, and writing. DH: analysis. ZH and OT: review and editing and supervision. All authors contributed to the article and approved the submitted version.

## Conflict of Interest

The authors declare that the research was conducted in the absence of any commercial or financial relationships that could be construed as a potential conflict of interest.

## Supplementary Material

Supplementary Figure S1 **(A)** Correlation analysis of ethanol production and relative abundance of *Bacteroides* in ND and HFD caecum. **(B)** Correlation analysis of ethanol production and relative abundance of *Bacteroides* in ND and HFD feces.

Supplementary Figure S2 **(A)** Correlation analysis of ethanol production and relative abundance of Proteobacteria in ND and HFD caecum. **(B)** Correlation analysis of ethanol production and relative abundance of Proteobacteria in ND and HFD feces.

Supplementary Figure S3 Change in bacterial population in terms of Log 10 CFU/ml was recorded at different time interval (6–24 h) during fermentation of fructooligosaccharide with **(A)** caecum microbiota **(B)** fecal microbiota. Results are means ± SEM (*n* = 3).

Supplementary Table S1 Composition of normal diet and high fat diet.
