# Supplementary figures and images for: High-Fat Diet Induced Alteration of Mice Microbiota and the Functional Ability to Utilize Fructooligosaccharide for Ethanol Production
Source: Front Cell Infect Microbiol. 2020 Aug 7;10:376. doi: 10.3389/fcimb.2020.00376 (PMC7426704; doi:10.3389/fcimb.2020.00376)

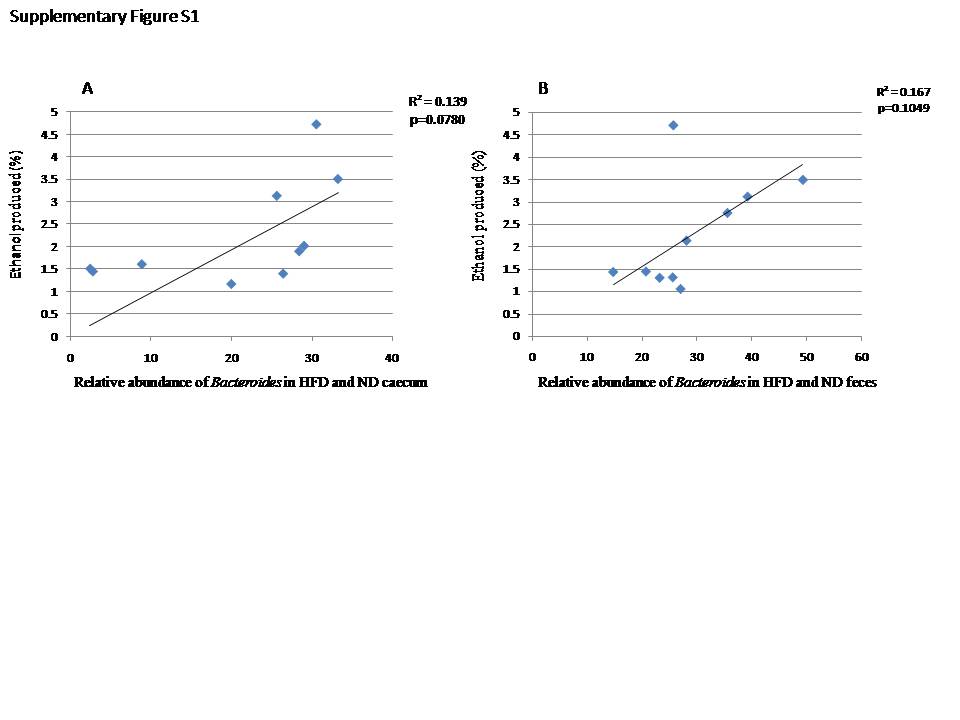

Supplement: Supplementary Figure S1 — (A) Correlation analysis of ethanol production and relative abundance of Bacteroides in ND and HFD caecum. (B) Correlation analysis of ethanol production and relative abundance of Bacteroides in ND and HFD feces. [file Image_1.JPEG]

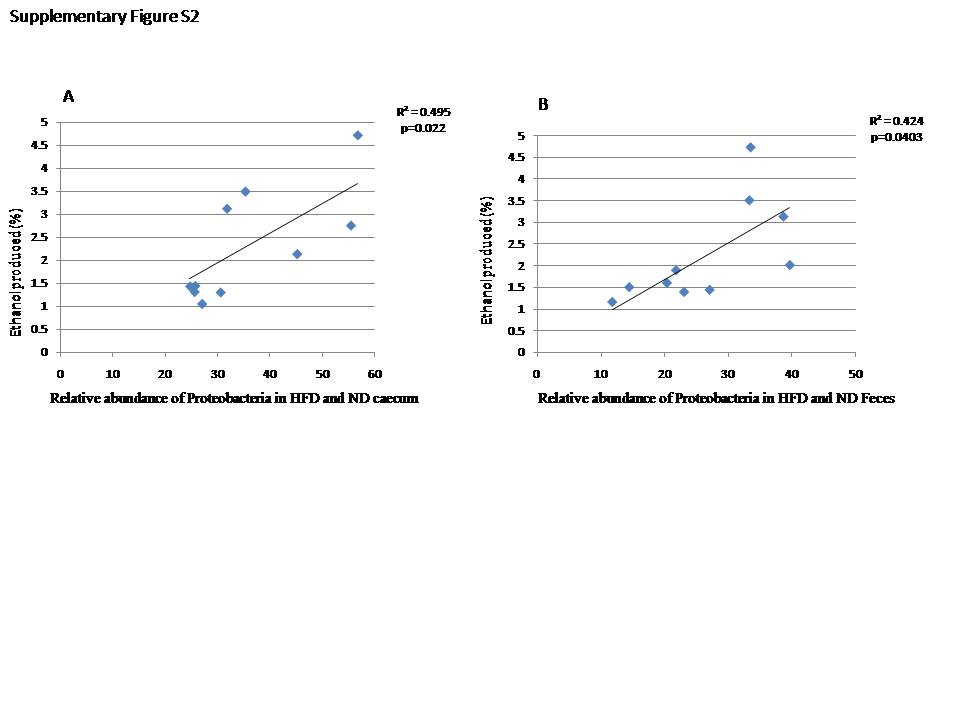

Supplement: Supplementary Figure S2 — (A) Correlation analysis of ethanol production and relative abundance of Proteobacteria in ND and HFD caecum. (B) Correlation analysis of ethanol production and relative abundance of Proteobacteria in ND and HFD feces. [file Image_2.JPEG]

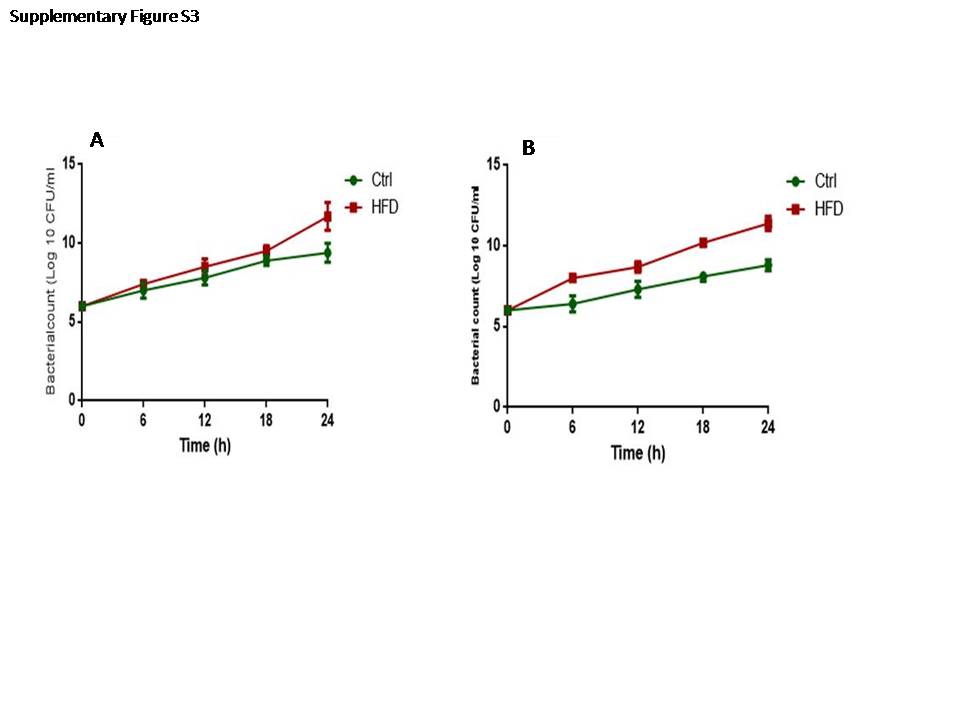

Supplement: Supplementary Figure S3 — Change in bacterial population in terms of Log 10 CFU/ml was recorded at different time interval (6–24 h) during fermentation of fructooligosaccharide with (A) caecum microbiota (B) fecal microbiota. Results are means ± SEM (n = 3). [file Image_3.JPEG]
