# Supplementary material for: High-Fat Diet Induced Alteration of Mice Microbiota and the Functional Ability to Utilize Fructooligosaccharide for Ethanol Production
Source: Front Cell Infect Microbiol. 2020 Aug 7;10:376. doi: 10.3389/fcimb.2020.00376 (PMC7426704; doi:10.3389/fcimb.2020.00376)
Supplement: Supplementary Table S1 — Composition of normal diet and high fat diet. [file Table_1.DOCX]

Supplementary Table S1. Composition of normal diet and high fat diet

| **Normal Diet (ND)** | **High fat diet (HFD)** |
| --- | --- |
| Corn starch (500g) | Cellulose (65.5g) |
| Casein (210g) | Casein (265g) |
| Maltodextrine (100g) | Maltodextrine (160g) |
| Sucrose (39.15g) | Sucrose (90g) |
| Butter (24g) | Cholic acid (5g) |
| Lard (20g) | Lard (310g) |
| Soyabeen oil (20g) | Soyabeen oil (30g) |
| Cellulose (35g) |  |
| Mineral mix (35g) | Mineral mix (51.4g) |
| Vitamin mix (15g) | Vitamin mix (21g) |
| L-methionine (3g) | L-methionine (4g) |
| Choline (2.75g) | Choline (5.0g) |
| BHT (0.014g) | BHT (0.014g) |
| DDW 500 ml | DDW 200 ml |
